# Practical Approach for the Management and Evaluation of Paraneoplastic Syndromes

**DOI:** 10.7759/cureus.11830

**Published:** 2020-12-01

**Authors:** Khalid Serraj, Siham Hamaz, Habiba Alaoui, Mohamed Barrimi, Ahmed Amine El Oumri

**Affiliations:** 1 Internal Medicine, Immunohematology and Cellular Therapy Laboratory, Faculty of Medicine and Pharmacy of Oujda, Mohammed First University of Oujda, Oujda, MAR; 2 Infectious Diseases, Immunohematology and Cellular Therapy Laboratory, Faculty of Medicine and Pharmacy of Oujda, Mohammed First University of Oujda, Oujda, MAR; 3 Immunohematology Cellular Therapy, Immunohematology and Cellular Therapy Laboratory, Faculty of Medicine and Pharmacy of Oujda, Mohammed First University of Oujda, Oujda, MAR; 4 immunohematology Cellular Therapy, Immunohematology and Cellular Therapy Laboratory, Faculty of Medicine and Pharmacy of Oujda, Mohammed First University of Oujda, Oujda, MAR

**Keywords:** malignancy, paraneoplastic syndromes, diagnosis, endocrine syndromes, nervous system, skin

## Abstract

Paraneoplastic syndromes (PNS) are conditions linked to the presence of tumors, most often malignant, without being the direct translation of a locoregional extension or distant metastases. They affect 10% to 15% of cancer patients, can appear before, after, or simultaneously with a cancer diagnosis, and primarily affect the nervous system, endocrine glands, and skin. The main tumors that provide PNS are lung cancer, gynecological tumors, and lymphomas. The diagnostic and therapeutic approaches are very heterogeneous due to the physiopathological specificities of each type of PNS. The main advances made in recent years have focused mainly on diagnostic tools, which have become more efficient in the diagnosis of PNS and underlying cancers.

## Introduction and background

The paraneoplastic syndrome (PNS) was first described by Auche in 1890 in a patient suffering from peripheral neuropathy. It has a broad nosological framework and is very common, found in 10% to 15% of cancer patients [1]. PNS is a condition related to the presence of a tumor, often malignant, without, however, being the direct translation of a locoregional or metastatic extension. PNS may occur earlier (60% of cases), concomitant, or subsequent to the diagnosis of cancer. According to the European Network of Cancer Registries, PNS is the second direct cause of death (27% of cases) after cancer itself. It is, therefore, essential to recognize and treat PNS quickly, energetically, and specifically. Bronchial tumors, gynecological cancers, and lymphomas are the main providers of PNS [1]. This article discusses the practical diagnostic approach and the therapeutic basics for PNS with the main focus on neurological, endocrine, and dermatological damage, as well as the main updates on inflammatory myopathies and venous thromboembolism.

## Review

Neurological paraneoplastic syndromes

**Figure 1:**
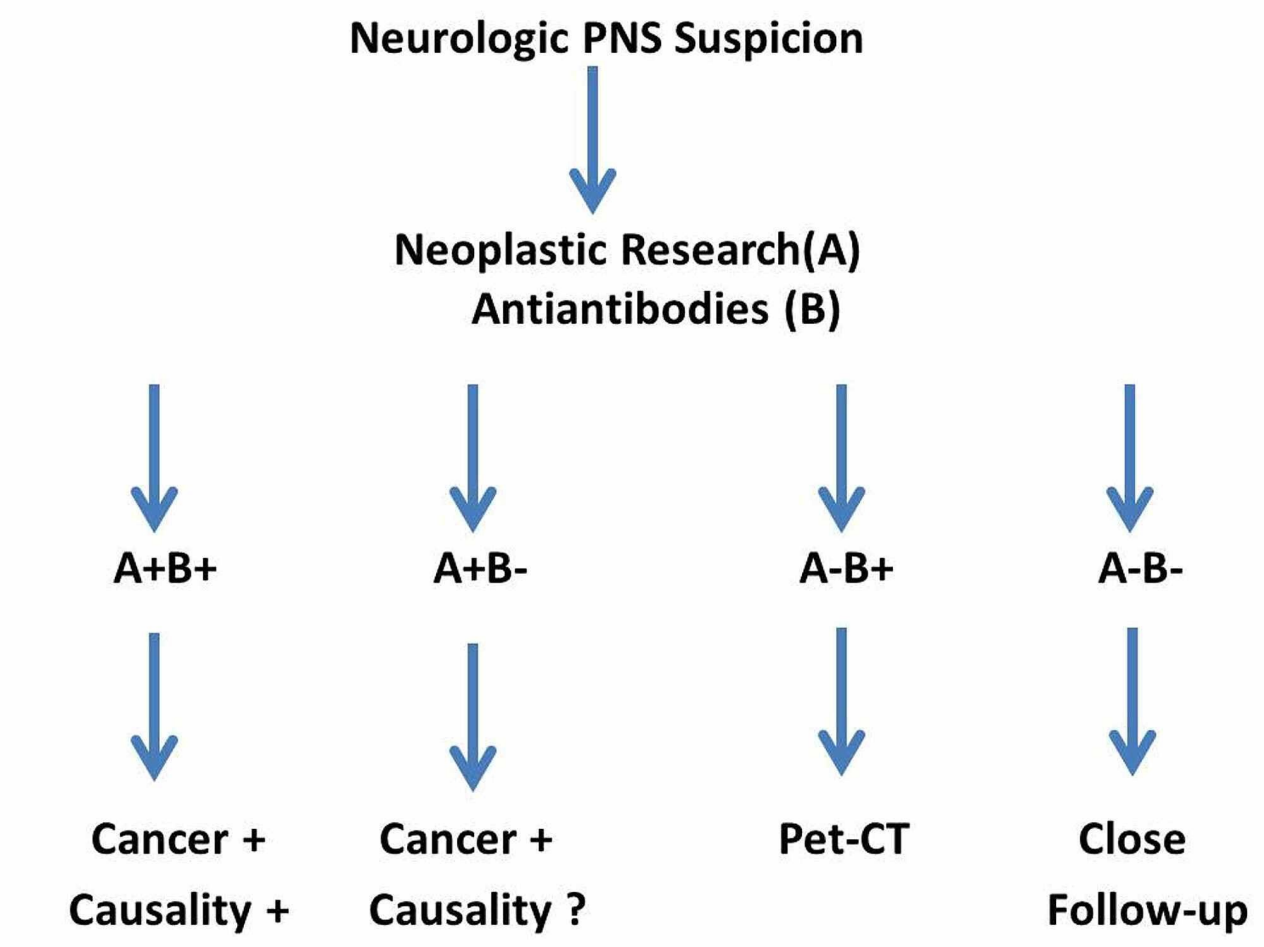
Diagnostic algorithm of neurologic PNS PNS: paraneoplastic syndromes

**Figure 2:**
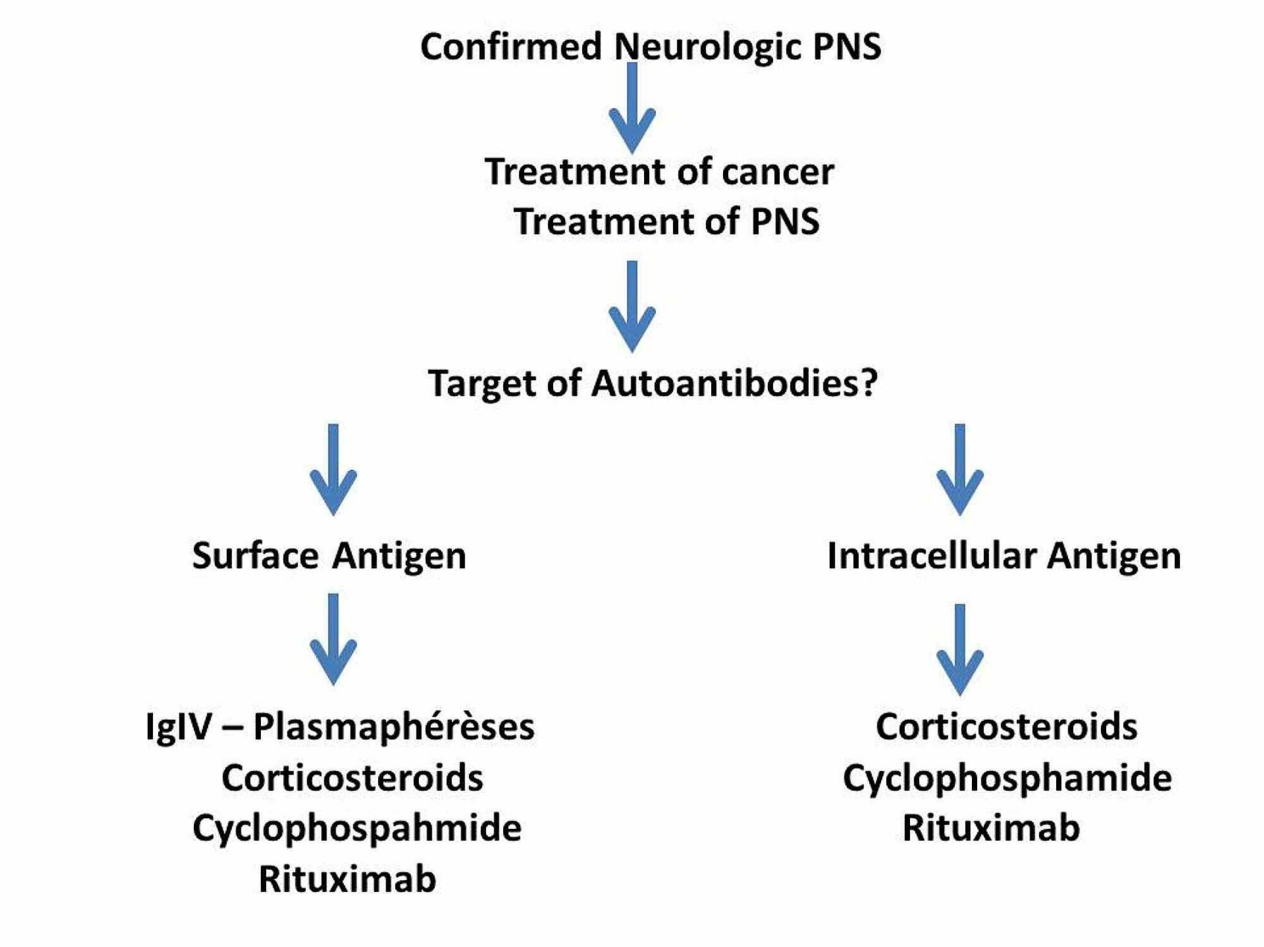
Management of neurologic PNS PNS: paraneoplastic syndromes

The main neurological PNS and their clinical, biological, and etiological characteristics are presented in Table 1. Their most common characteristics are the acute or subacute course and the lymphocytic pleocytosis of the cerebrospinal fluid (CSF), often revealing a previously occult cancer. The main challenge is, therefore, to know when to suspect and how to confirm PNS and then to identify the underlying neoplasm. The dysimmunity is the main pathophysiological mechanism of neurological PNS. Indeed, specific autoantibodies are found in more than half of cases and are increasingly used as diagnostic tools. Consequently, the concomitant research of antibodies and underlying cancer allows the diagnosis of both PNS and tumor with no delay between the two conditions (Figure 1). Therapeutically, immunosuppressive molecules and/or immunomodulators must usually be associated with anticancer drugs, and the modalities of their use are now increasingly guided by the neuronal, intracytoplasmic, or membrane tropism of the identified autoantibodies. Indeed, an antibody targeting a surface antigen is assumed to be more exposed at the plasma level and would be more accessible to plasmapheresis and immunoglobulins. In contrast, intracellular antibodies, less exposed, would be much less sensitive to plasma exchange and immunoglobulins than classical immunosuppressants (Figure 2) [2].

**Table 1:** Pathogenesis and causes of neurologic PNS PNS: paraneoplastic syndromes

Neurologic PNS	Pathogenesis and Autoantibodies	Underlying Cancer
Encephalomyelitis	T cytotoxic Autoimmunity Humoral Autoimmunity (Anti-Hu)	Small Cells, Lung Cancer
Limbic Encephalitis	Humoral Autoimmunity (Anti-Hu, Ma, CV)	Small Cells, Lung Cancer, Testicle
Anti-R-NMDA Encephalitis	Humoral Autoimmunity anti-R-NMDA	Ovarian Teratoma
Brainstem Encephalitis	Unknown	Lung, Breast, Colon, Parotid
Opsoclonus – Myoclonus	Humoral Autoimmunity Anti-Neurofilaments Humoral Autoimmunity Anti-RI	Lung, Breast, Neuroblastoma
Cerebellous Degenerescence	Humoral Autoimmunity Anti-Purkinje cells	Lung, Breast, Ovaries, Hodgkin
Lambert-Eaton Syndrome	Autoimmunity Anti-Voltage Gated Calcic Canals (VGCC)	Small Cells, Lung Cancer
Peripheral Neuropathy	Unknown	Lung, Breast, Lymphoma
Vasculitis	Unknown	Lymphoma
Autonomic Neuropathy	Unknown	Small Cells, Lung Cancer
Neuromyotonia	Unknown	Small Cells, Lung Cancer, Thymoma

Endocrine paraneoplastic syndromes

Table 2 summarizes the main endocrine PNS and their characteristics.

**Table 2:** Pathogenesis and causes of endocrine PNS PNS: paraneoplastic syndromes; PTHrp: parathyroid hormone-related protein; TGF: transforming growth factor; TNF: tumor necrosis factor; IL: interleukin; ADH: antidiuretic hormone; ACTH: adrenocorticotropic hormone

Endocrine paraneoplastic syndrome	Pathogenesis	Underlying cancer
Inappropriate ADH Secretion	Anti-Diuretic Hormone	Lung, Brain
Hypercalcemia	PTHrp, TGF, TNF, IL1	Myeloma, Lung, Breast, Ovaries, Kidney, Lymphoma
Hypoglycemia	Insulin, Insulin-Like	Liver Sarcoma
Cushing Syndrome	ACTH, ACTH-Like	Neuroendocrine Tumors, Lung, Thymoma, Pancreas
Carcinoïd Syndrome	Sérotonin, Bradykinin	Carcinoïd Tumors, Pancreas. Stomach

Endocrine PNS are the result of the secretion by the tumor of a hormone, prohormone, or other substance that has a pathological effect on healthy tissues. Unlike neurological PNS, the occurrence of endocrine PNS classically happens during the course of an already identified cancer. The diagnostic challenge is, therefore, not the same, as the causality is generally easily established. Moreover, the parallelism between the intensity of PNS and the stage of the tumor is rarely established. Therapeutically, the goal in addition to anti-tumor drugs is to neutralize the tissue effects of the secreted substance. The main endocrine PNS symptoms are hypercalcemia, paraneoplastic crumbs, hyponatremia by the inappropriate secretion of antidiuretic hormone, paraneoplastic hypo and hyperglycemia, osteomalacia, Cushing's syndrome, carcinoid syndrome, and Zollinger-Elison syndrome. For all these entities, and in the absence of immunological markers, the diagnostic approach is based on classical algorithms in front of clinical and/or biological revealing abnormalities [3].

Cutaneous paraneoplastic syndromes

More than 30 cutaneous PNS have been recognized to date [4]. It is necessary to distinguish between cutaneous paraneoplastic syndromes and skin metastases or local infiltration of a primary tumor. The pathophysiology is often ambiguous and very hypothetical. The roles of growth factors, metabolic abnormalities due to the tumor, and immunological mechanisms (cross-reaction between tumor and skin antigens) are discussed. Some syndromes are pathognomonic and should be considered paraneoplastic until proven otherwise. Unlike neurological and endocrine PNS, the diagnosis and causality of cutaneous PNS are much more based on the clinical and visual experience of the physician. A skin biopsy is only of interest for a positive diagnosis of the suspected lesion and, by definition, does not demonstrate the presence of tumor cells. Digestive tumors are the main providers of cutaneous PNS, and the treatment is essentially based on the management of underlying cancer [4].

Other paraneoplastic syndromes

Rheumatologic PNS are quite common, led by inflammatory myopathies (IM), which are associated with cancer in 10% to 20% of cases. The most important progress in recent years in IM has been the recognition of autoimmune necrotizing myositis (ANS) as an independent entity as well as dermatomyositis, polymyositis, and inclusion myositis. This is an affection exhibiting the clinical, electromyographical, and biological characteristics of inflammatory myopathy with, as the main particularities: a history of taking statins, the presence of specific anti-hydroxymethyl-glutaryl-coenzyme A reductase (anti-HMGCR) autoantibodies, the absence of inflammatory lymphoplasmacytic infiltrate muscle biopsy, and frequent resistance to corticosteroids alone [5]. The main other rheumatologic PNS are very similar to the clinical presentations of Still's disease, rhizomelic pseudopolyarthritis, and edematous polyarthritis of the elderly. In practice, any seronegative joint damage in an elderly male must suggest the presence of underlying malignancy especially if there is significant weight loss [6].

The association of thrombosis and cancer is now widely documented and confirmed (20% to 50% of cases) [7-8]. Cancer screening in any patient with thrombosis is systematically recommended, as well as thromboprophylaxis in cancer patients, along with several curative specificities for thrombosis in the context of cancer. In this regard, low molecular weight heparins (LMWH) are the treatment of choice for the entire duration of anticoagulation due to their superiority to anti-vitamin K in the prevention of thrombotic recurrences and their hypothetical antineoplastic effects. Recently, the non-inferiority of some direct oral anticoagulants (DOAC) like edoxaban and rivaroxaban in cancer-related thromboprophylaxis has been demonstrated in comparison with LMWH. However, gastrointestinal bleeding is more frequent with DOAC, which are, therefore, avoided in patients with digestive malignancy [7]. Table 3 and Table 4 summarize the main cutaneous, rheumatological, hematological, and vascular PNS [4,6,8].

**Table 3:** Pathogenesis and causes of rheumatologic and dermatologic PNS PNS: paraneoplastic syndromes; VEGF: vascular endothelial growth factor; PGE2: prostaglandin E_2_; TGF: transforming growth factor; EGF: epidermal growth factor

Paraneoplastic Syndrome	Pathogenesis	Underlying Cancer
Polymyositis	T Cytotoxic Autoimmunity	Lung
Dermatomyositis	Humoral Autoimmunity and Vasculitis	Breast, Ovaries, Prostate
Hypertrophic Osteoarthropathy	VEGF, PGE2	Lung
Acanthosis Nigricans	TGF, EGF	Stomach, Lung, Uterus
Sweet Syndrome	Neutrophilic Dermatosis	Myeloproliferative Diseases, Breast, Digestive Malignancy
Pemphigus	Cross-Immunity with Skin	B Lymphoma
Exfoliating Erythroderma	Unknown	T Lymphoma, Bladder, Colon, Rectum

**Table 4:** Pathogenesis and causes of hematological PNS PNS: paraneoplastic syndromes; VEGF: vascular endothelial growth factor; IL: interleukin; EPO: erythropoietin; GM-CSF: granulocyte-macrophage colony-stimulating factor; G-CSF: granulocyte colony-stimulating factor

Paraneoplastic syndrome	Pathogenesis	Underlying cancer
Leukocytoclastic Vasculitis	Unknown	Lymphoma, Lung, Stomach, Urologic Malignancies
Deep Vein Thrombosis	Factor X Tissular Factor, Platelet Activation, Entholelial Cells, Activation VEGF	Pancreas, Kidney, Digestive Cancers, All Types of Cancer
Polycythemia	Erythropoïétin EPO-Like	Kidney, Liver, Uterus, Cerebellous Tumors
Thrombocytosis	IL-6	Lung, Digestive Cancers
Eosinophilia	IL-1 IL-2, IL-5, GM-CSF	Lung, Digestive Cancers, Gynecologic Cancers
Neutrophilia	G-CSF	Lung, Digestive Cancers, Kidney, Brain, Gynecologic Cancers
Erythroblastopenia	T Cytotoxic Autoimmunity	Lymphoma, Thymoma

What to do in case of negative neoplastic assessment?

The search for occult neoplasia justifies the usefulness of the positron emission tomography (PET) scan when classical biology, imaging, and morphological examinations are unremarkable. In a study by the Mayo Clinic, the PET scan revealed the presence of cancer in 40% of patients with suspected PNS and normal CT scan. In cases of negative assessment, clinical monitoring is required for two to five years among asymptomatic patients, and a paraclinical assessment should be realized whenever there are signs in favor of a recurrence of the initial symptomatology, especially if no other etiological differential diagnosis has been identified [9].

## Conclusions

PNS are very challenging for clinicians in both their diagnostic and their therapeutic particularities. The methodical application of diagnostic algorithms is necessary in order to shorten the time between the appearance of a PNS and the moment of its positive diagnosis and therapeutic management.* *These algorithms must be pertinent, and the management mostly based on a good understanding of the pathophysiological mechanisms of PNS and rapid anti-tumoral therapy.
